# Combining Molecular Docking and Molecular Dynamics to Predict the Binding Modes of Flavonoid Derivatives with the Neuraminidase of the 2009 H1N1 Influenza A Virus

**DOI:** 10.3390/ijms13044496

**Published:** 2012-04-10

**Authors:** Shih-Jen Lu, Fok-Ching Chong

**Affiliations:** 1 Department of Electrical Engineering, National Taiwan University, Taipei 10617, Taiwan; E-Mail: fcchong931@hotmail.com; 2 Department of Research and Development, BroadMaster Biotech Co., Ltd.: 7F., No.168-2, Liancheng Rd., Zhonghe Dist., New Taipei City 23553, Taiwan

**Keywords:** molecular dynamics, H1N1, neuraminidase, molecular docking

## Abstract

Control of flavonoid derivatives inhibitors release through the inhibition of neuraminidase has been identified as a potential target for the treatment of H1N1 influenza disease. We have employed molecular dynamics simulation techniques to optimize the 2009 H1N1 influenza neuraminidase X-ray crystal structure. Molecular docking of the compounds revealed the possible binding mode. Our molecular dynamics simulations combined with the solvated interaction energies technique was applied to predict the docking models of the inhibitors in the binding pocket of the H1N1 influenza neuraminidase. In the simulations, the correlation of the predicted and experimental binding free energies of all 20 flavonoid derivatives inhibitors is satisfactory, as indicated by *R*^2^ = 0.75.

## 1. Introduction

Since March 2009, a new strain of the influenza A virus (H1N1) has rapidly spread to many countries from the initial outbreak in South America. In July 2009, the WHO (World Health Organization) declared that the spread of H1N1 influenza virus had become a serious global pandemic. Recently, the H1N1 influenza associated reports state that several mutation strains of H1N1 influenza A viruses are resistant to oseltamivir and zanamivir [1–3]. Influenza viruses have been classified by the antigenic properties of two glycoproteins, sixteen hemagglutinin proteins and nine neuraminidase proteins [4,5]. The hemagglutinin proteins play a role as antigens binding to the sialic acid receptor on the host cell surface, which aids the entry of the virus [6]. The function of the neuraminidase antigen is to cleave the terminal linkage of the sialic acid receptor, which results in the release of the progeny virions from the infected host cells. In addition, neuraminidase may have a function as importer facilitating the early process of the infection of lung epithelial cells by the influenza virus [7]. Because of its essential role in influenza virus replication and its highly conserved active sites, neuraminidase has been an attractive target for the development of novel anti-influenza drugs [8–14].

In recent years, several flavonoids have been reported as showing anti-influenza virus activity by inhibiting neuraminidase [15–19]. Flavonoids are low molecular weight compounds that are widespread in the plant kingdom. Those compounds have been shown to possess several biological effects in mammals [20–22]. In particular *in vitro* studies, scientists report that flavonoids may inhibit several enzymes related to the cardiovascular system [23–27], especially inhibiting the Matrix Metalloproteinases (MMPs) [28]. Therefore, using flavonoids as antivirals should be carefully considered in addition to these other proposed activities. In general, flavonoids are interesting molecules combining an aromatic nature with several hydrophilic groups. These aromatic interactions play a key role in protein-protein and protein-ligand interactions [29–31]. The hydrophilic nature (hydroxyl (OH) functional group of flavonoids/water molecules) of the falvonoids shows that water displacement is key for determining ligand affinity [32–36].

Scientists also report that the flavonoid derivatives can efficiently inhibit the activity of H1N1 neuraminidase [37]. To reveal the inhibition mechanism of flavonoid derivatives on H1N1 neuraminidase, a knowledge of the three-dimensional structure of H1N1 neuraminidase is indispensable. Since H1N1 neuraminidase structures have been determined by X-ray experiments [5,38], we chose the structure (PBD ID: 3NSS) as the target structure for these studies.

In this study, the 20 flavonoid derivatives (2,3-dihydrobenzofuran and 5,7-dihydroxychromen-4-one backbones) and their experimental biological binding affinities [37,39] were chosen to simulate H1N1 neuraminidase pharmacological activities; these inhibitors are listed in Table S1. The transfer function [40] (Δ*G*_bind_ = −*RT* ln(IC_50_)) is used to transfer the experimental values (IC_50_ ) to the experimental binding free energies values; these experimental values are listed in Table S1. Molecular docking, molecular dynamics simulations (MD), and binding free energies calculations were used to gain further insight into the binding interactions between the 2009 H1N1 neuraminidase and the 20 flavonoid derivatives inhibitors.

## 2. Results and Discussion

### 2.1. Molecular Docking and MD Simulation

**Figure 1:**
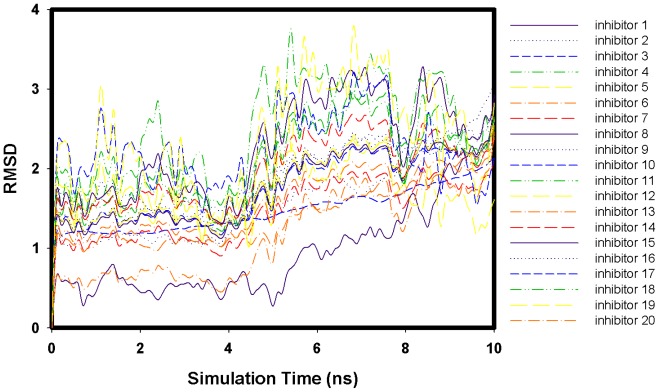
RMSD profiles of 20 flavonoid derivatives.

**Figure 2:**
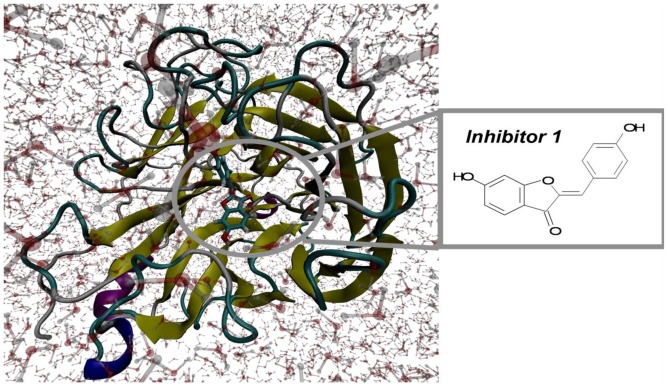
The snapshot of the 2009 H1N1 neuraminidase of the inhibitor 1.

The 20 flavonoid derivatives were docked into the H1N1 neuraminidase structure. Over the 10-ns MD trajectories of the H1N1 neuraminidase with tip3 water molecules and flavonoid derivatives, the overall structure of both complexes appeared to be equilibrated after 324 ps. Here, we show the RMSD profiles of 20 flavonoid derivatives (Figure 1) and the snapshot (Figure 2) of the complex system of the flavonoid derivatives **1**. The RMSD values of 20 flavonoids stay within 4 Å.

### 2.2. Key Residues of 2009 H1N1 Neuraminidase

The study of these 20 compounds has revealed that the amino residues can frequently interact with flavonoid inhibitors in the H1N1 neuraminidase binding site, and that these residues are responsible for the selectivity of flavonoid inhibitors. The results of our simulations are listed in Table 1 and Figure S1–S20. The inhibitors 1–3 and 14 (Table 1) belong to the 2,3-dihydrobenzofuran backbone inhibitors and the others belong to the 5,7-dihydroxychromen-4-one backbone inhibitors. In the 2,3-dihydrobenzofuran backbone inhibitors (inhibitor 1–3 and 14), Asn295, Glu119, Glu277, Thr226, Trp179 can form hydrogen bonds in the 2009 H1N1 neuraminidase/flavonoids complex structures and Asn295 most frequently forms the hydrogen bonds. Only Tyr402 has non-bonding interactions with inhibitor 1 (Figure S1). In the 5,7-dihydroxychromen-4-one backbone inhibitors (inhibitor 4–13 and 15–20), Arg152, Asn295, Asn325, Asn344, Asp151, Asp294, Glu119, Glu228, Glu277, Ser180, Ser247, Ser366, Ser367, Thr226, Trp179, Tyr402 and Val346 can form hydrogen bonds in the complex structures and Glu228 most frequently forms the hydrogen bonds. Arg368, Ile223, Pro326 and Trp179 have non-bonding interactions with the backbone inhibitors (Figure S7, 16 and 19). The overall results of our simulations suggest that Arg152, Asn295, Asn325, Asn344, Asp151, Asp295, Glu119, Glu228, Glu277, Ser180, Ser247, Ser366, Ser367, Thr226, Trp179, Tyr402 and Val346 can form hydrogen bonds between the 2009 H1N1 neuraminidase and flavonoid derivatives. Moreover, our simulations indicate that Arg368, Ile223, Pro326 and Trp179 have non-bonding interactions with these derivatives. The non-bonding interactions of the 2009 H1N1 neuraminidase/flavonoid complex structures only occurred in inhibitor 1, 7, 16 and 19 simulations. While six residues (Arg152, Asn295, Glu228, Glu277 Trp179 and Val346) more often formed the hydrogen bonds of the complex structures, Asn295 most frequently formed the hydrogen bonds.

### 2.3. Flavonoid Derivatives Binding Free Energies

**Figure 3:**
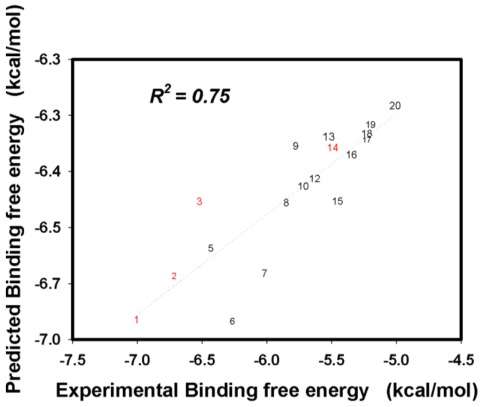
Predicted binding free energies *versus* experimentally determined binding free energies of the 20 inhibitors. The correlation constant (*R*^2^) is equal to 0.75. The inhibitors 1–3 and 14 (red) belong to the 2,3-dihydrobenzofuran backbone inhibitors and the others belong to the 5,7-dihydroxychromen-4-one backbone inhibitors (black).

The 2009 H1N1 neuraminidase/flavonoid (inhibitors 1–20) complex structures and the Tip3 water solvent box were constructed using the Autodock Vina docking and AmberTools 1.5 programs. The binding free energies of each inhibitor were obtained from the 10-ns MD simulation and the SIE method, with both processes using the same parameters. All the results are listed in Table 2. In the simulations, the predicted binding free energies of the 20 inhibitors was in good agreement with the experimental results (Figure 3), with the correlation coefficient being 0.75.

### 2.4. Free Energies Contribution Analysis of Hydrophobic and Hydrophilic Nature of 20 Flavonoids

The hydrophobic and hydrophilic nature of 20 flavonoids was individually traced down the binding affinities with the important residue regions analyzed by the ligplot program (Arg152, Asn295, Asn325, Asn344, Asp151, Asp295, Glu119, Glu228, Glu277, Ser180, Ser247, Ser366, Ser367, Thr226, Trp179, Tyr402), and the water molecules (within a 10 Å radius of 20 flavonoids). All the results are listed in Tables S2,3 and Figures S21,22. For the hydrophobic nature analysis (aromatic groups), the two residues (Trp179 and Tyr402) have obvious binding affinities to the inhibitors. The contributions to Trp179 and Tyr402 binding free energies are −1.121~−2.961 and −1.665~−3.143 kcal/mol, respectively. For the hydrophilic nature analysis, the three elements (side chain of nitrogen atoms/Arg152, side chain of nitrogen and oxygen atoms/Asn295 and the water molecules within 10 Å of inhibitors) have obvious binding affinities to the inhibitors. Those contributions to Arg152, Asn295 and water molecules binding free energies are −0.507~−2.733, −0.185~−2.247 and −0.6~−2.6 kcal/mol, respectively. Figure S23 shows that the more hydroxyl function groups are present, the higher the binding affinities of the inhibitors.

## 3. Materials and Methods

### 3.1. Molecular Docking

All flavonoid derivatives inhibitors were constructed and minimized using VEGA ZZ [41] and ISIS/DRAW [42] programs. We aligned the H1N1 neuraminidase structure (PBD ID: 3NSS) and the template (PDB ID: 3B7E) [43], and then used the template structure drug (zanamivir) as a template to generate the active site of the H1N1 neuraminidase structure. Next, the Autodock vina docking program [44] was used to dock the 20 flavonoid derivatives inhibitors into the active site of the H1N1 neuraminidase structure, which was defined as all residues within 0.15 nm from alignment with the template structure drug. Autodock vina is a fast and accurate way to dock small compounds into fixed protein binding sites, utilizing NNscore [45] and several types of genetic algorithms. Four thousand conformations were obtained from docking for the 20 inhibitors, and these then scored by NNscore. The conformations of the best NNscores were then selected for subsequent MD simulations.

### 3.2. Molecular Dynamics Simulation

Calculations were performed with the NAMD molecular dynamics software [46] using the AMBER FF99 all-hydrogen amino acid and general amber force field (GAFF) parameters. The GAFF partial atomic charges are often based on the RESP fitting procedure of the electrostatic potential obtained at the HF/6–31G(d,p) level of theory [47]. Those levels of theory overestimate the gas-phase partial atomic charges giving rise to an effectively polarized force field. The geometries of the 20 flavonoid derivatives inhibitors were fully optimized and their electrostatic potentials were obtained using a single-point calculation; both operations were carried out at the HF level with the 6–31 G(d,p) basis set using the US GAMESS [48] program. Subsequently, their partial charges were obtained by the restrained electrostatic potential (RESP) using R.E.D tools [49]. From the docking simulations, the complex structures were inserted into the tip3p water box. All MD simulations were performed in the NVT ensemble (temperature equal to 310 K), unless noted, using a Verlet integrator, an integration time step of 0.002 ps, and SHAKE [50] data for all covalent bonds involving hydrogen atoms. In electrostatic interactions, atom-based truncation was undertaken using the PME method. In addition, the switch van der Waals functions were also used with a 1.8 nm cutoff for atom-pair lists. The complex structures were minimized for 20,000 conjugate gradient steps. The minimized complex structures were then subjected to a 10 ns isothermal, constant volume MD simulation. The MD simulation trajectories were converted to the Amber type coordinates by the AmberTools 1.5 program [51]. All MD simulation results were used to initiate the functionally important residues and the binding free energies calculations.

### 3.3. Functionally Important Residues of H1N1 Influenza Neuraminidase

In a diseased target proteins, not every residue is equally important. Some residues are essential for important enzymatic functions and protein structure stability. Thus identification of functionally important residues can provide a clear insight into the structural aspects of H1N1 2009 influenza neuraminidase. In this work, the structure-based approach was applied to identify functionally important residues, while MD simulations were used to identify the residues involved in the binding pocket. In the MD simulations, the important residues regions were analyzed by the ligplot program [52]. The results of functional important residues were applied to binding free energies calculations.

### 3.4. Binding Free Energies Calculations (Solvated Interaction Energies Method)

The binding free energies calculations were performed by the solvated interaction energies method (SIE). The binding free energies between receptor and inhibitors were calculated for snapshot structures taken from the MD trajectory of the system. From the 10 ns protein-ligand MD trajectories, 100 snapshots were taken at regular intervals for the binding energies analyses. The SIE function [53] to estimate the binding free energies is written as:

(1) $$\begin{array}{l} \Delta G_{bind}(\rho ,D_{in},\alpha ,\gamma ,C)=\alpha \times [E_{c}(D_{in})+\Delta G_{bind}^{R}(\rho ,D_{in})\\ +E_{vdw}+\gamma \Delta MSA(\rho )]+C \end{array}$$

where *E_C_* and *E_vdw_* are the intermolecular Coulomb and van der Waals interaction energies in the bound state, respectively. These values were calculated using the AMBER molecular mechanics force field (FF99) with an optimized dielectric constant. Δ*G_bind_^R^* is the change in the reaction field energies between the bound and free states and is calculated by solving the Poisson equation with the boundary element method program, BRI BEM, and using a molecular surface generated with a variable-radius solvent probe. The ΔMSA term is the change in the molecular surface area upon binding. The following parameters are calibrated by fitting to the absolute binding free energies for a set of protein–ligand complexes: AMBER van der Waals radii linear scaling coefficient (*ρ*), the solute interior dielectric constant (Din), the molecular surface area coefficient (*γ*), the global proportionality coefficient related to the loss of configurational entropy upon binding (*α*), and a constant (*C*). The optimized values of these parameters are *α* = 0.1048, Din = 2.25, *ρ* = 1.1, *γ* = 0.0129 kcal/(mol Å^2^), and *C* = −2.89 kcal/mol. The SIE calculations were carried out with the program sietraj [53]. The hydrophobic (non-hydroxyl group) and hydrophilic (hydroxyl group) nature of flavonoids can affect the binding abilities [29–31]. Thus these natures were individually traced down to the binding affinities with the whole H1N1 neuraminidase, the important residue regions analyzed by the ligplot program (hydrophilic and hydrophobic parts listed in Table S4), and the water molecules (within a 10 Å radius of 20 flavonoids).

## 4. Conclusions

In this article, we used the Autodock vina program, tip3 water solvent model, MD simulations techniques, and the SIE method to predict the binding modes in which a series of flavonoid derivatives inhibitors interact with the 2009 H1N1 neuraminidase. From our simulations results, the correlation coefficient between the predicted binding free energies and experimental values of the 20 inhibitors is equal to 0.75. In the 2,3-dihydrobenzofuran backbone derivatives inhibitors (inhibitor 1–3 and 14), Asn295 forms the hydrogen bonds most frequently. In the 5,7-dihydroxychromen-4-one backbone derivatives inhibitors (inhibitor 4–13 and 15–20), Glu228 forms the hydrogen bonds most frequently.

The overall results of our simulations indicate that Arg152, Asn295, Asn325, Asn344, Asp151, Asp294, Glu119, Glu228, Glu277, Ser180, Ser247, Ser366, Ser367, Thr226, Trp179, Tyr402 and Val346 can form hydrogen bonds between the 2009 H1N1 neuraminidase and flavonoid derivatives. While six residues (Arg152, Asn295, Glu228, Glu277 Trp179 and Val346) more often form the hydrogen bonds of the complex structures, Asn295 forms the hydrogen bonds most frequently. In the free energies contribution analysis, the two residues (the indole group of Trp179 and the benzene group of Tyr402) have obvious binding affinities to the hydrophobic nature (aromatic-aromatic interactions) of the 20 inhibitors ((2Z)-2-benzylidene-3H-benzofuran, 2-phenyl-4H-chromene and benzene aromatic groups), and the three elements (the guanidine group of Arg152, the acetamide group of Asn295 and the water molecules within 10 Å of inhibitors) have obvious binding affinities to the hydrophilic nature of the 20 inhibitors (–OH and =O groups). Figure S23 also shows that the more hydroxyl and oxygen function groups are present, the greater the number of binding affinities of the inhibitors. Therefore, our approach theoretically suggests that the four residues Arg152, Trp179, Asn295 and Tyr402 are responsible for the selectivity of the flavonoid derivatives.

## Supplementary Information



## Figures and Tables

**Table 1:** Important results: Important residues of the 2009 H1N1 neuraminidase from the molecular docking and molecular dynamics (MD) simulations.

Inhibitors	Hydrogen bonding–related residues	Non-bonding contact-related residues
1(A)	Trp179, Thr226, Asn295	Tyr402
2(A)	Glu277, Asn295	Null
3(A)	Glu119, Asn295	Null
4(B)	Glu119, Ser180, Glu228, Asp294, Val346	Null
5(B)	Val346	Null
6(B)	Arg152, Glu228, Glu277 Asn295	Null
7(B)	Asn295	Trp179, Ile223
8(B)	Glu119, Ser180, Glu228, Asp294, Val346	Null
9(B)	Arg152, Glu228, Val346	Null
10(B)	Ser180, Thr226, Glu228, Asp294	Null
11(B)	Glu228, Glu277	Null
12(B)	Asp151, Glu277, Asn325, Val346, Ser366	Null
13(B)	Trp179, Asn295	Null
14(A)	Asn295	Null
15(B)	Arg152, Trp179, Asn295	Null
16(B)	Glu119, Asp151, Ser366, Ser367, Tyr402	Ile223, Arg368
17(B)	Trp179	Null
18(B)	Asp151, Arg152, Asn344	Null
19(B)	Asp151, Trp179, Glu228, Asn295	Pro326
20(B)	Arg152, Trp179, Ser247, Glu277	Null

**A**: 2,3-dihydrobenzofuran backbone and **B**: 5,7-dihydroxychromen-4-one backbone.

**Table 2:** Binding free energies for the 2009 H1N1 neuraminidase/flavonoid (inhibitors 1–20) complex structures by the SIE method.

Inhibitors	Energy (kcal/mol)					
	
	*E_C_*	*E_vwd_*	Δ*MSA*	Δ*G_bind_^R^*	Δ*G_bind_* (SIE)	Δ*G_bind_* (Experiment)
**1**	−20.7	−9.95	6.12	−6.98	−6.825	−7.002
**2**	−20.9	−7.36	5.29	−5.64	−6.435	−6.714
**3**	−15.2	−6.69	4.83	−5.71	−5.771	−6.518
**4**	−20.3	−5.31	5.31	−5.91	−6.186	−6.430
**5**	−16.9	−6.12	5.81	−7.41	−6.071	−6.296
**6**	−24.1	−7.26	6.43	−6.39	−6.837	−6.265
**7**	−20.9	−7.36	5.11	−5.43	−6.413	−6.014
**8**	−16.9	−4.81	6.57	−5.94	−5.779	−5.847
**9**	−12.9	−4.64	5.11	−5.33	−5.277	−5.773
**10**	−15.4	−4.73	5.59	−6.11	−5.633	−5.717
**11**	−13.9	−4.91	6.19	−6.82	−5.567	−5.629
**12**	−12.2	−6.71	3.91	−6.08	−5.503	−5.608
**13**	−11.4	−5.73	4.41	−4.91	−5.193	−5.519
**14**	−12.4	−5.12	5.25	−5.51	−5.291	−5.489
**15**	−15.4	−4.73	5.65	−6.41	−5.766	−5.450
**16**	−9.3	−8.73	6.81	−5.56	−5.353	−5.343
**17**	−12.9	−4.32	4.81	−5.09	−5.221	−5.226
**18**	−12.7	−4.21	4.61	−4.84	−5.163	−5.225
**19**	−11.6	−4.29	4.82	−5.11	−5.084	−5.199
**20**	−10.1	−4.33	4.99	−4.97	−4.916	−5.007
