# Supplementary material for: Combining Molecular Docking and Molecular Dynamics to Predict the Binding Modes of Flavonoid Derivatives with the Neuraminidase of the 2009 H1N1 Influenza A Virus
Source: Int J Mol Sci. 2012 Apr 10;13(4):4496–507. doi: 10.3390/ijms13044496 (PMC3344228; doi:10.3390/ijms13044496)
Supplement: Supplementary file 1 [file ijms-13-04496-s001.pdf]

Supplementary Materials

Table S1. 20 flavonoids derivatives inhibitors of the 2009 H1N1 neuraminidase.

**Inhibitors**<br>Hydrophilic (blue color) and hydrophobic (red color) parts for SIE and RDF calculations	**Experimental**<br>**Binding free energy** ΔG_b^{Exp} **(kcal/mol) and Experimental** IC_{50} **(mM)**
**1:** (2Z)-6-hydroxy-2-[(4-hydroxyphenyl)methylene]benzofuran-3-one<br>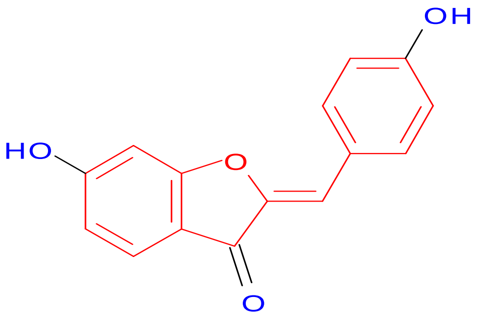	−7.002<br>(0.00559)
**2:** (2Z)-4,6-dihydroxy-2-[(4-hydroxyphenyl)methylene]benzofuran-3-one<br>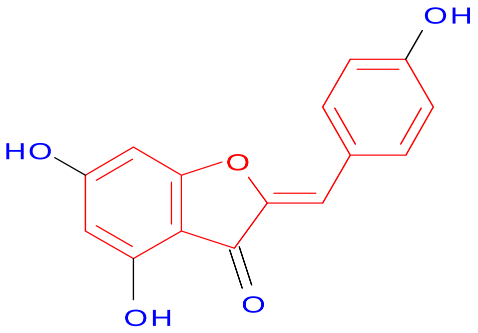	−6.714<br>(0.00692)
**3:** (2Z)-2-[(3,4-dihydroxyphenyl)methylene]-6-hydroxy-benzofuran-3-one<br>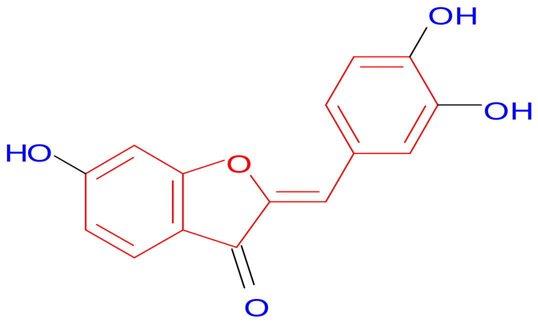	−6.518<br>(0.00800)

Table S1. *Cont.*

**Inhibitors**<br>**Hydrophilic (blue color) and hydrophobic (red color) parts for SIE and RDF calculations**	**Experimental**<br>**Binding free energy ΔG_b^{Exp} (kcal/mol) and Experimental IC_{50} (mM)**
**4:** 5,7-dihydroxy-2-(4-hydroxyphenyl)chromen-4-one	
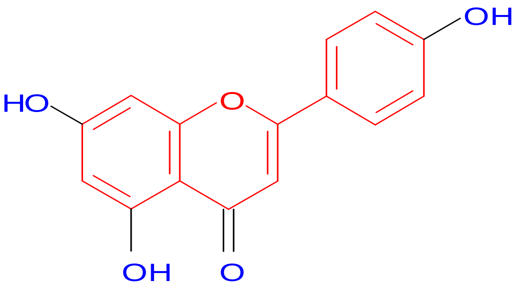	−6.430<br>(0.00854)
**5:** 7-hydroxy-3-(4-hydroxyphenyl)chromen-4-one	
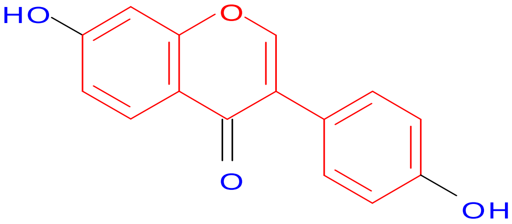	−6.296<br>(0.00943)
**6:** 2-(3,4-dihydroxyphenyl)-5,7-dihydroxy-chromen-4-one	
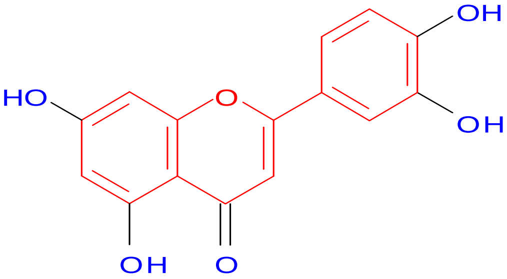	−6.265<br>(0.00965)
**7:** 5,7-dihydroxy-2-phenyl-chromen-4-one	
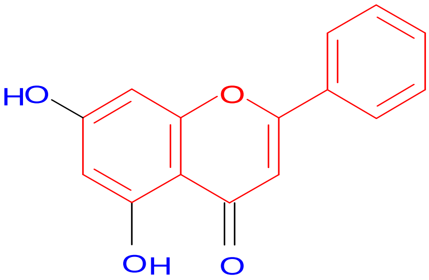	−6.014<br>(0.01162)

Table S1. *Cont.*

**Inhibitors**<br>**Hydrophilic (blue color) and hydrophobic (red color) parts for SIE and RDF calculations**	**Experimental**<br>**Binding free energy ΔG_b^{Exp} (kcal/mol) and Experimental IC_{50} (mM)**
**8:** 5,7-dihydroxy-2-(4-hydroxyphenyl)-8-methoxy-chromen-4-one<br>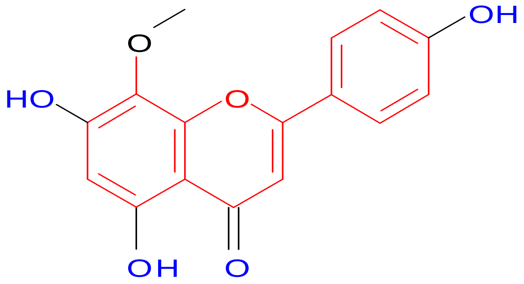 	−5.847<br>(0.01315)
**9:** 5,7-dihydroxy-2-(4-hydroxyphenyl)-6-methoxy-chromen-4-one<br>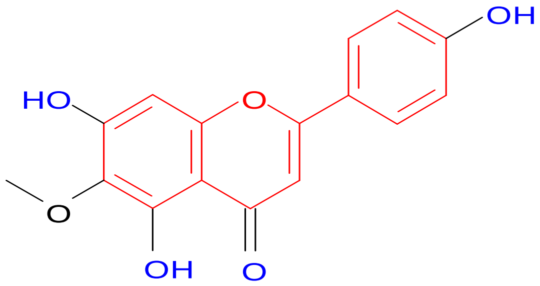 	−5.773<br>(0.01390)
**10:** 5,6,7-trihydroxy-2-(4-hydroxyphenyl)chromen-4-one<br>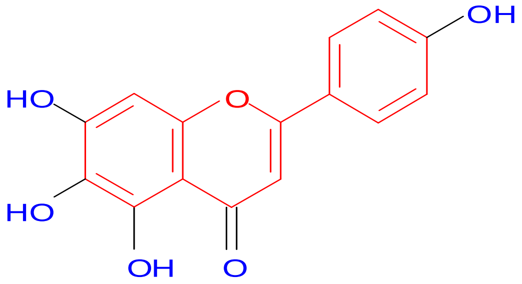 	−5.717<br>(0.01448)
**11:** 5,6,7-trihydroxy-2-(4-hydroxyphenyl)chromen-4-one<br>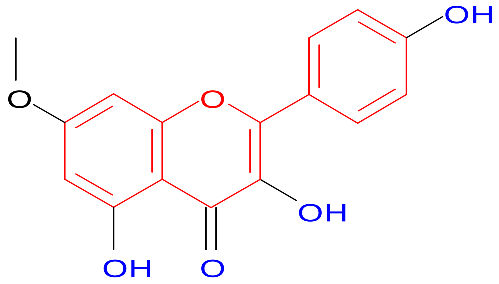 	−5.629<br>(0.01546)

Table S1. *Cont.*

**Inhibitors** **Hydrophilic (blue color) and hydrophobic (red color) parts for SIE and RDF calculations**	**Experimental** **Binding free energy ΔG_b^{Exp} (kcal/mol) and Experimental IC_{50} (mM)**
**12:** [5-hydroxy-2-(3,4,5-trihydroxyphenyl)chroman-7-yl] 3,4,5-trihydroxybenzoate 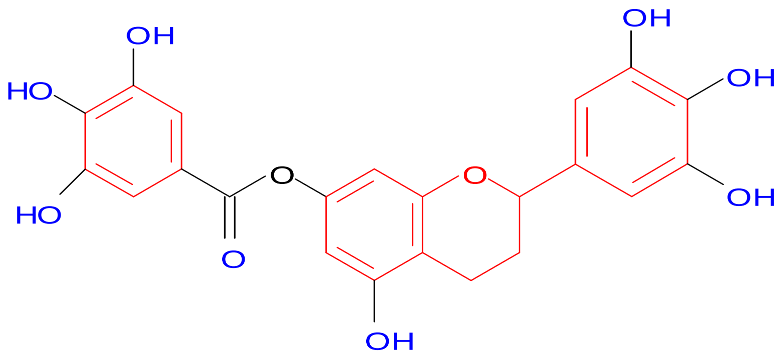	−5.608 (0.15700)
**13:** 3,5,7-trihydroxy-2-(4-hydroxyphenyl)chromen-4-one 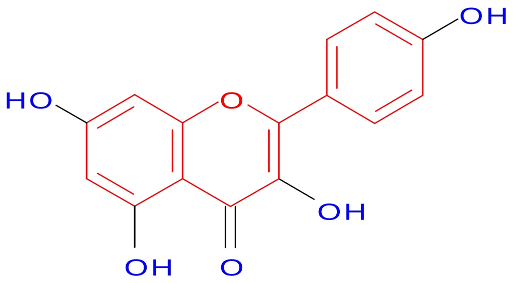	−5.519 (0.01677)
**14:** (2E)-2-benzylidene-6-hydroxy-benzofuran-3-one 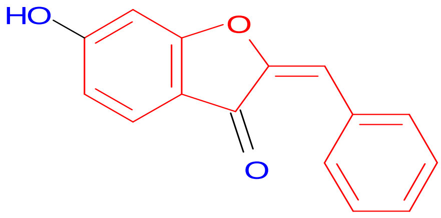	−5.489 (0.01715)
**15:** 2-(3,4-dihydroxyphenyl)-3,5,7-trihydroxy-chromen-4-one 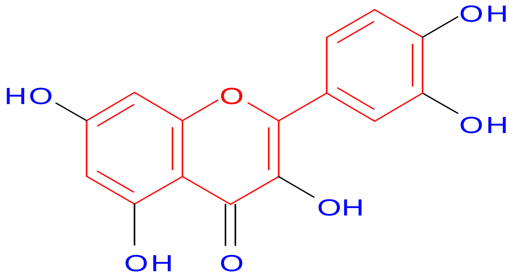	−5.450 (0.01765)

Table S1. *Cont.*

**Inhibitors**<br>**Hydrophilic (blue color) and hydrophobic (red color) parts for SIE and RDF calculations**	**Experimental**<br>**Binding free energy ΔG_b^{Exp} (kcal/mol) and Experimental IC_{50} (mM)**
**16:** 5-acetamido-4-hydroxy-2-[5-hydroxy-6-[4-(5-hydroxy-7-methoxy-4-oxo-chromen-2-yl)phenoxy]-2-(4-methoxyphenyl)-4-oxo-chromen-7-yl]oxy-6-(1,2,3-trihydroxypro pyl)tetrahydropyran-2-carboxylic acid<br>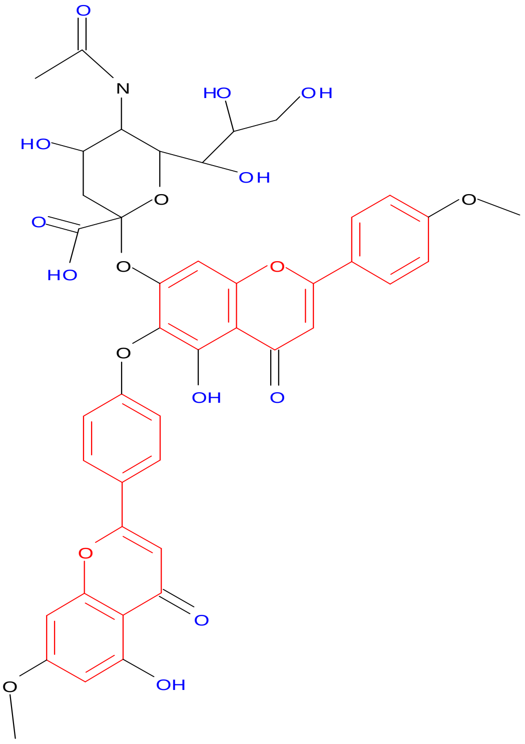	−5.343<br>(0.01910)
**17:** 5,7-dihydroxy-3-(4-hydroxyphenyl)chromen-4-one<br>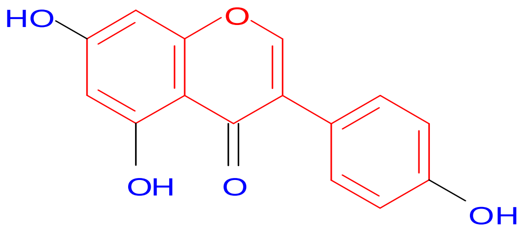	−5.226<br>(0.02084)

Table S1. *Cont.*

**Inhibitors**<br>**Hydrophilic (blue color) and hydrophobic (red color) parts for SIE and RDF calculations**	**Experimental**<br>**Binding free energy ΔG_b^{Exp} (kcal/mol) and Experimental IC_{50} (mM)**
**18:** 5,7-dihydroxy-2-(4-hydroxyphenyl)-8-[3,4,5-trihydroxy-6-(hydroxymethyl)tetrahydropyran-2-yl]chromen-4-one<br>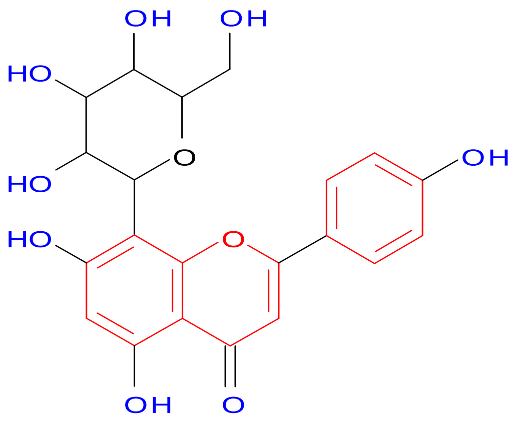 	−5.450<br>(0.02085)
**19:** 2-(3,4-dihydroxyphenyl)-5-hydroxy-7-[3,4,5-trihydroxy-6-(hydroxymethyl)tetrahydropyran-2-yl]oxy-chromen-4-one<br>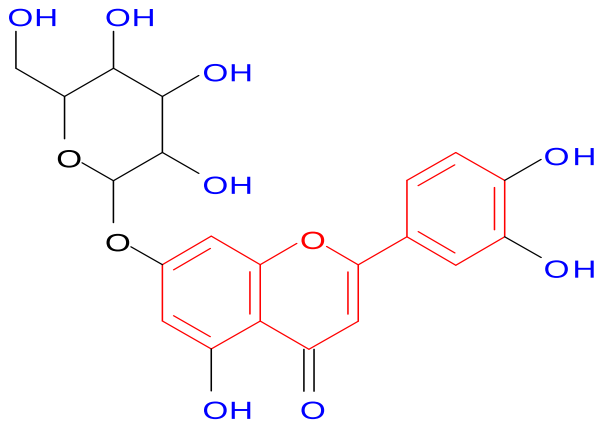 	−5.163<br>(0.02125)

Table S1. *Cont.*

**Inhibitors**<br>**Hydrophilic (blue color) and hydrophobic (red color) parts for SIE and RDF calculations**	**Experimental**<br>**Binding free energy ΔG_b^{Exp} (kcal/mol) and Experimental IC_{50} (mM)**
**20:** 3-[4,5-dihydroxy-6-(hydroxymethyl)-3-(3,4,5-trihydroxytetrahydropyran-2-yl)oxy-tetrahydropyran-2-yl]oxy-5,7-dihydroxy-2-(4-hydroxyphenyl)chromen-4-one<br>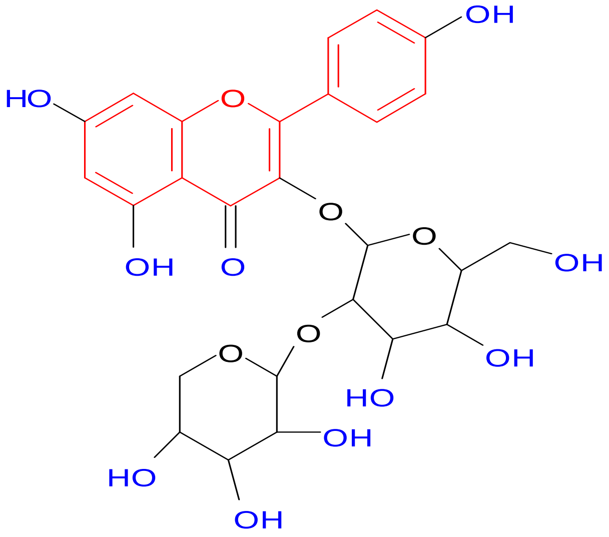	−5.084<br>(0.02450)

Table S2. Binding free energies analysis of hydrophobic natures of 20 flavonoids/hydrophobic natures of the important residue regions analyzed by the ligplot program (kcal/mol).

**Inhibitor**	**1**	**2**	**3**	**4**	**5**	**6**	**7**	**8**	**9**	**10**	**11**	**12**	**13**	**14**	**15**	**16**	**17**	**18**	**19**	**20**
Arg152	0	0	0	0	0	0	0	0	0	0	0	0	0	0	0	0	0	0	0	0
Asn295	0	0	0	0	0	0	0	0	0	0	0	0	0	0	0	0	0	0	0	0
Asn325	0	0	0	0	0	0	0	0	0	0	0	0	0	0	0	0	0	0	0	0
Asn344	0	0	0	0	0	0	0	0	0	0	0	0	0	0	0	0	0	0	0	0
Asp151	0	0	0	0	0	0	0	0	0	0	0	0	0	0	0	0	0	0	0	0
Asp294	0	0	0	0	0	0	0	0	0	0	0	0	0	0	0	0	0	0	0	0
Glu119	0	0	0	0	0	0	0	0	0	0	0	0	0	0	0	0	0	0	0	0
Glu228	0	0	0	0	0	0	0	0	0	0	0	0	0	0	0	0	0	0	0	0
Glu277	0	0	0	0	0	0	0	0	0	0	0	0	0	0	0	0	0	0	0	0
Ser180	0	0	0	0	0	0	0	0	0	0	0	0	0	0	0	0	0	0	0	0
Ser247	0	0	0	0	0	0	0	0	0	0	0	0	0	0	0	0	0	0	0	0
Ser366	0	0	0	0	0	0	0	0	0	0	0	0	0	0	0	0	0	0	0	0
Ser367	0	0	0	0	0	0	0	0	0	0	0	0	0	0	0	0	0	0	0	0
Thr226	0	0	0	0	0	0	0	0	0	0	0	0	0	0	0	0	0	0	0	0
Trp179	−2.348	−1.941	−1.985	−1.958	−2.067	−1.835	−2.419	−2.128	−1.854	−1.965	−1.761	−2.769	−1.803	−1.121	−1.922	−2.961	−2.151	−1.996	−1.856	−1.712
Tyr402	−1.944	−2.178	−1.978	−2.665	−2.588	−2.145	−1.718	−1.179	−2.114	−1.611	−2.021	−3.143	−1.607	−1.287	−1.744	−2.872	−1.951	−1.853	−1.706	−1.665
Val346	0	0	0	0	0	0	0	0	0	0	0	0	0	0	0	0	0	0	0	0
TIP3 water	0	0	0	0	0	0	0	0	0	0	0	0	0	0	0	0	0	0	0	0

Table S3. Binding free energies analysis of hydrophilic nature of 20 flavonoids/hydrophilic nature of natures of the important residue regions analyzed by the ligplot program (kcal/mol).

Inhibitor	1	2	3	4	5	6	7	8	9	10	11	12	13	14	15	16	17	18	19	20
Arg152	−0.56	−0.722	−0.507	−1.334	−0.154	−1.896	−1.11	−1.506	−0.868	−1.248	−1.587	−1.624	−1.761	−0.72	−1.105	−1.561	−0.601	−2.252	−2.409	−2.733
Asn295	−0.378	−0.46	−0.185	−0.172	−0.012	−0.514	−0.768	−0.524	−0.426	−0.506	−0.885	−2.247	−1.239	−0.538	−1.534	−1.321	−0.649	−1.871	−2.169	−2.333
Asn325	−0.174	−0.19	−0.082	−0.25	0.054	−0.134	−0.086	−0.214	−0.1	−0.15	−0.302	−0.61	−0.254	−0.166	−0.294	−0.794	−0.234	−0.502	−0.814	−0.846
Asn344	−0.199	−0.255	0.073	−0.515	−0.125	−0.359	−0.191	−0.239	−0.034	−0.355	−0.487	−0.988	−0.419	−0.011	−0.619	−0.803	−0.044	−0.965	−0.756	−0.868
Asp151	−0.031	−0.065	−0.073	−0.43	−0.034	−0.546	−0.144	−0.316	−0.202	−0.55	−0.473	−0.765	−0.171	−0.034	−0.286	−1.636	−0.196	−0.458	−0.621	−0.689
Asp295	−0.546	−0.556	−0.176	−0.281	0.059	−0.021	−0.491	−0.071	−0.292	−0.001	−0.096	−0.649	−0.566	−0.361	−0.621	−1.571	−0.371	−0.404	−0.599	−0.619
Glu119	−0.138	−0.196	−0.292	−0.901	−0.049	−0.993	−0.119	−0.883	−0.145	−0.391	−0.542	−1.446	−0.187	−0.128	−0.362	−1.377	−0.017	−1.012	−1.442	−1.558
Glu228	−0.04	−0.086	−0.138	−0.621	0.003	−0.725	−0.087	−0.555	−0.129	−0.741	−0.778	−1.326	−0.24	−0.037	−0.385	−1.119	−0.06	-0.948	−1.144	−1.236
Glu277	−0.142	−0.176	−0.184	−0.541	−0.145	−0.657	−0.255	−0.427	−0.313	−0.661	−0.584	−1.176	−0.282	−0.145	−0.397	−1.332	0.108	−0.654	−1.317	−1.385
Ser180	−0.044	−0.066	−0.03	−0.261	−0.093	−0.389	−0.223	−0.099	−0.297	−0.381	−0.19	−0.826	−0.124	−0.053	−0.209	−0.859	−0.139	−0.375	−0.804	−0.848
Ser247	-0.098	−0.11	−0.154	−0.28	−0.052	−0.268	−0.032	−0.328	−0.016	−0.28	−0.394	−0.75	−0.158	-0.092	−0.188	−0.888	−0.168	−0.694	−0.928	−0.952
Ser366	−0.319	-0.325	−0.097	−0.16	0.104	−0.004	−0.286	−0.034	0.152	0.02	−0.037	−0.815	−0.319	−0.136	−0.364	−0.814	−0.154	−0.622	−0.739	−0.751
Ser367	−0.227	−0.231	−0.079	−0.121	−0.045	−0.017	−0.205	−0.037	0.067	−0.021	−0.059	−0.711	−0.247	−0.225	−0.257	−0.757	−0.217	−0.659	−0.737	−0.745
Thr226	−0.292	−0.294	−0.218	−0.239	−0.051	−0.187	−0.281	−0.197	−0.115	−0.159	−0.178	−0.804	−0.272	−0.111	−0.307	−0.857	−0.137	−0.763	−0.802	−0.806
Trp179	0	0	0	0	0	0	0	0	0	0	0	0	0	0	0	0	0	0	0	0
Tyr402	−0.352	−0.354	−0.278	−0.299	−0.111	−0.247	−0.341	−0.257	−0.235	−0.279	−0.298	−0.824	−0.392	−0.231	−0.367	−0.917	−0.197	−0.913	−0.952	−0.956
Val346	0	0	0	0	0	0	0	0	0	0	0	0	0	0	0	0	0	0	0	0
Tip3	−0.614	−0.685	−0.603	−0.689	−0.645	−0.879	−0.513	−0.613	−0.615	−0.7606	−0.654	−2.577	−0.516	−0.2261	−0.878	−1.861	−0.135	−1.824	−1.713	−1.864

Table S4. Hydrophilic and hydrophobic natures of the important residue regions analyzed by the ligplot program.

**The important residue regions analyzed by the ligplot program**	**Hydrophilic (blue color) and hydrophobic (red color) parts for SIE and RDF calculation**
Arg152	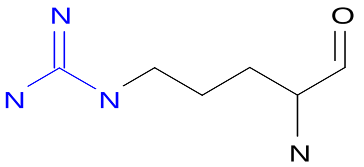
Asn295, Asn325 and Asn344	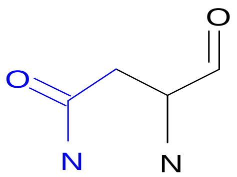
Asp151 and Asp295,	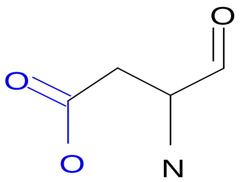
Glu119, Glu228 and Glu277,	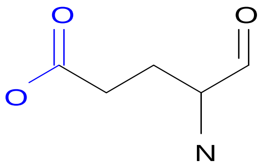
Ser180, Ser247, Ser366 and Ser367,	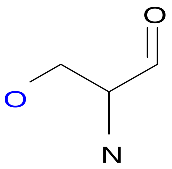
Thr226	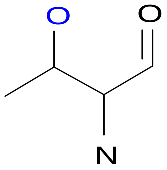
Trp179	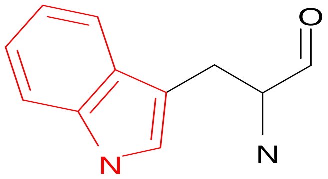
Tyr402	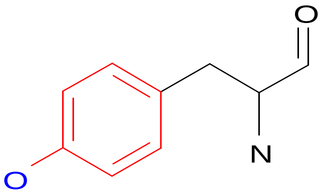

Table S4. *Cont.*

The important residue regions analyzed by the ligplot program	Hydrophilic (blue color) and hydrophobic (red color) parts for SIE and RDF calculation
Val346	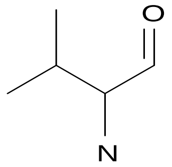

Figure S1. (**A**) Pharmacophore of Inhibitor 1; (**B**) Inhibitor 1 bound to the binding site of the 2009 H1N1 neuraminidase.

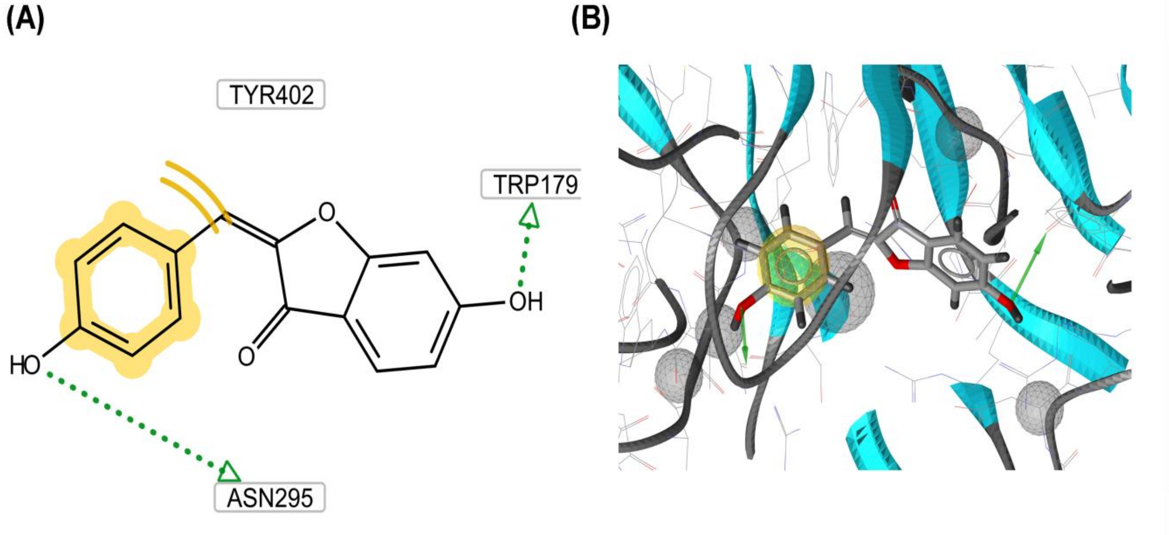

Figure S2. (**A**) Pharmacophore of Inhibitor 2; (**B**) Inhibitor 2 bound to the binding site of the 2009 H1N1 neuraminidase.

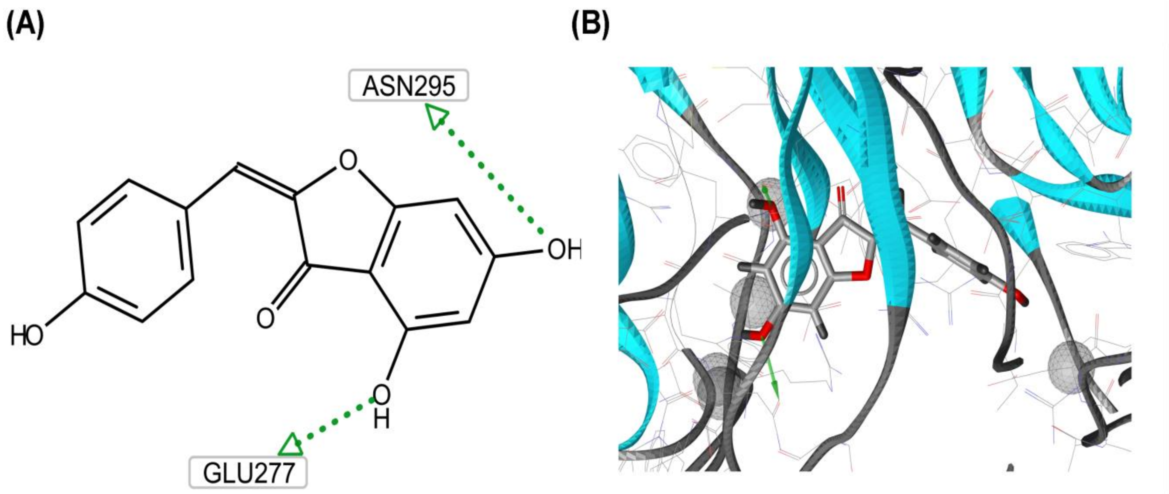

Figure S3. (**A**) Pharmacophore of Inhibitor 3; (**B**) Inhibitor 3 bound to the binding site of the 2009 H1N1 neuraminidase.

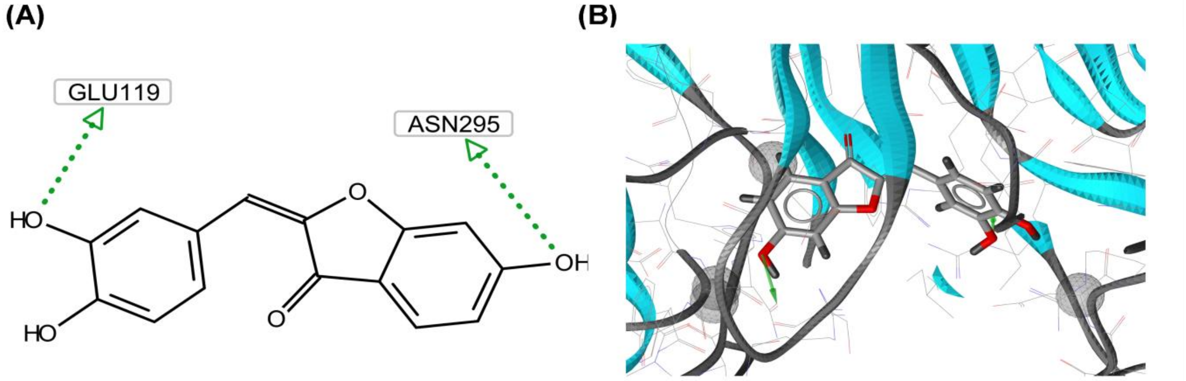

Figure S4. (**A**) Pharmacophore of Inhibitor 4; (**B**) Inhibitor 4 bound to the binding site of the 2009 H1N1 neuraminidase.

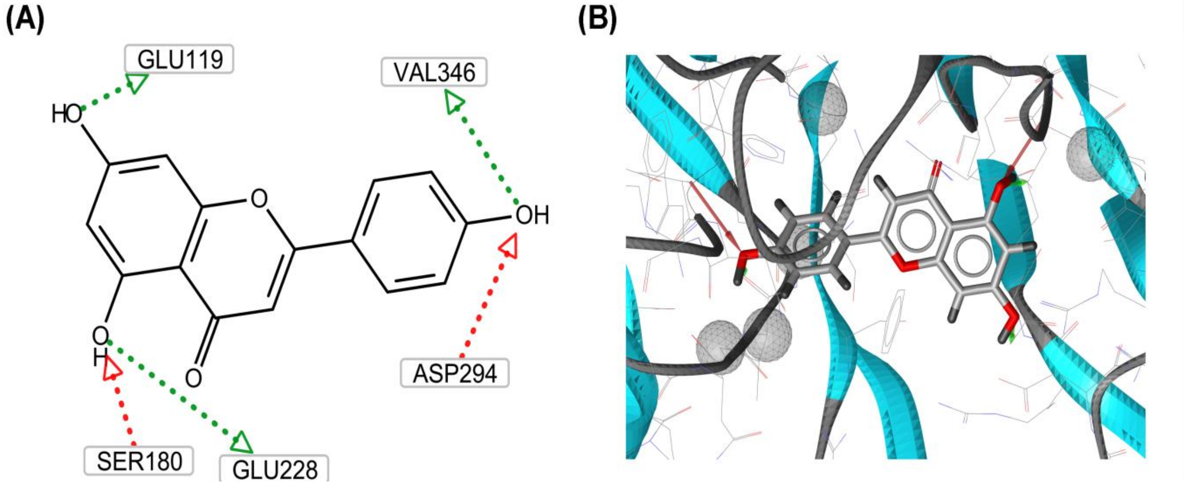

Figure S5. (**A**) Pharmacophore of Inhibitor 5; (**B**) Inhibitor 5 bound to the binding site of the 2009 H1N1 neuraminidase.

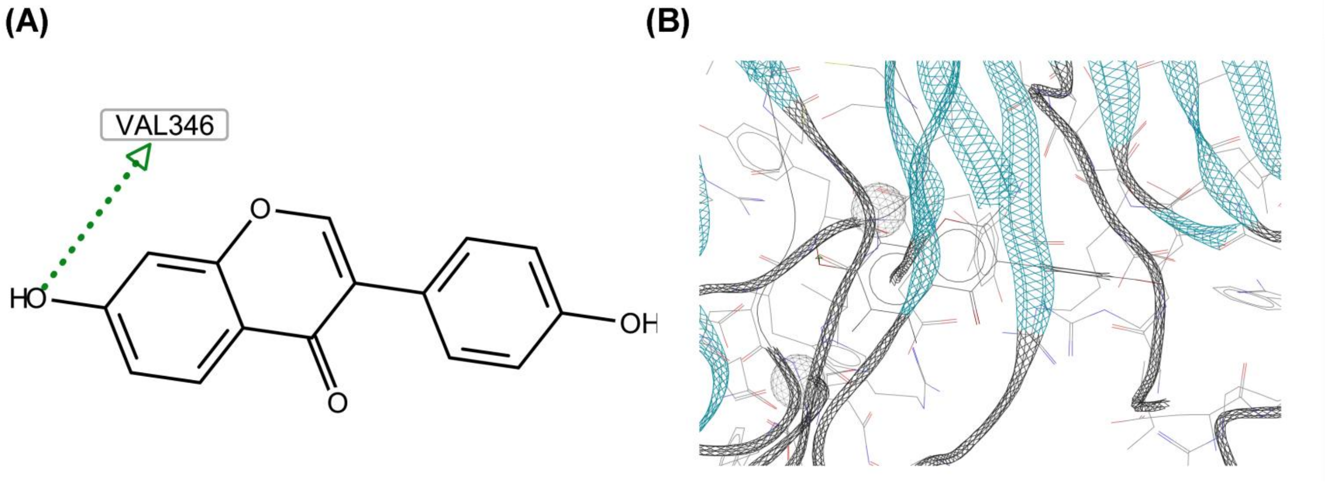

Figure S6. (**A**) Pharmacophore of Inhibitor 6; (**B**) Inhibitor 6 bound to the binding site of the 2009 H1N1 neuraminidase.

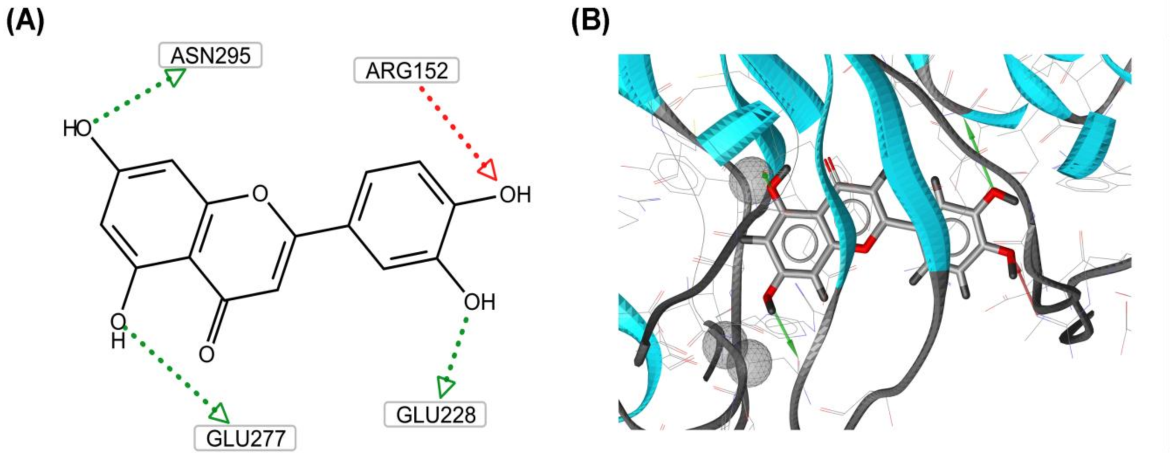

Figure S7. (**A**) Pharmacophore of Inhibitor 7; (**B**) Inhibitor 7 bound to the binding site of the 2009 H1N1 neuraminidase.

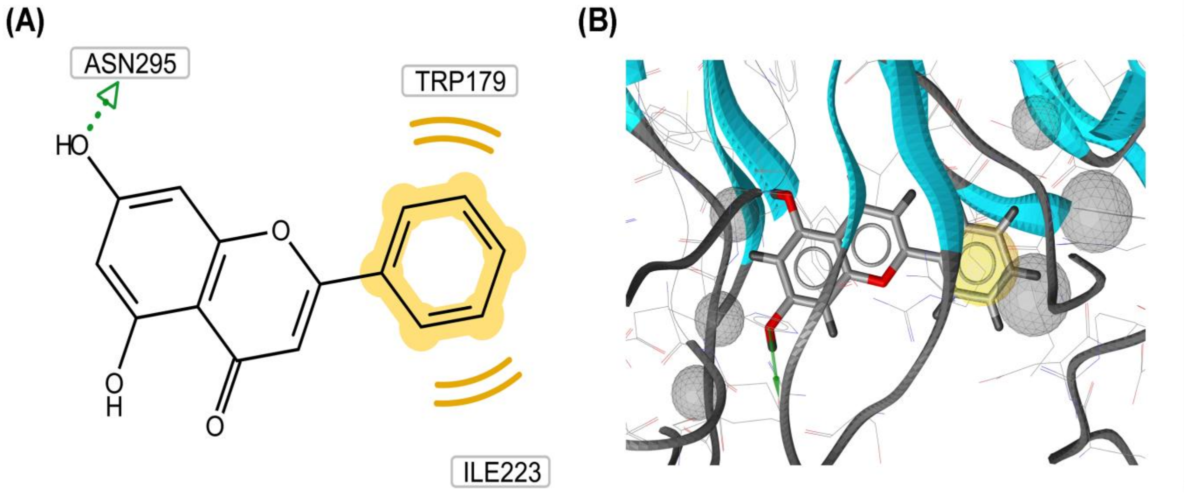

Figure S8. (**A**) Pharmacophore of Inhibitor 8; (**B**) Inhibitor 8 bound to the binding site of the 2009 H1N1 neuraminidase.

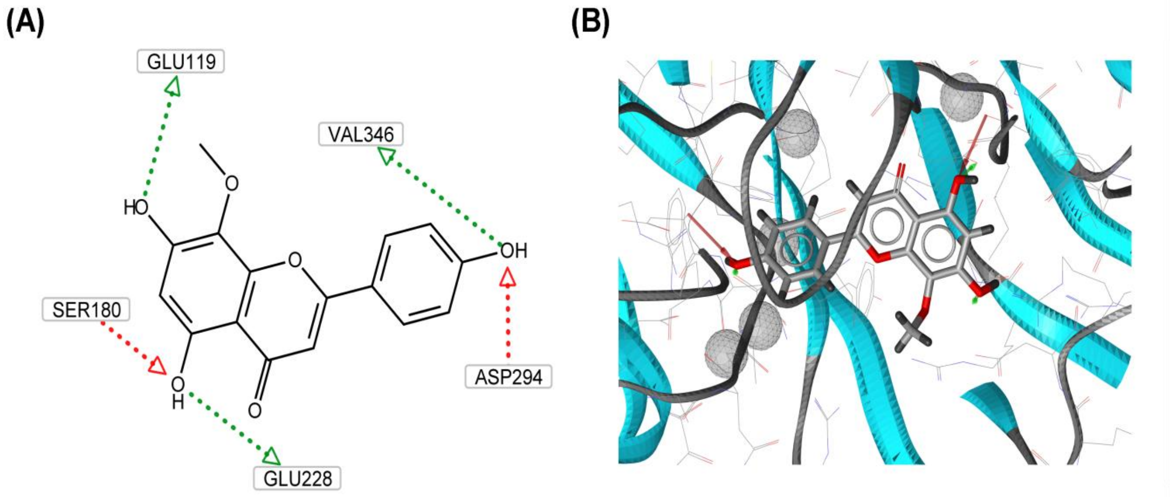

Figure S9. (**A**) Pharmacophore of Inhibitor 9; (**B**) Inhibitor 9 bound to the binding site of the 2009 H1N1 neuraminidase.

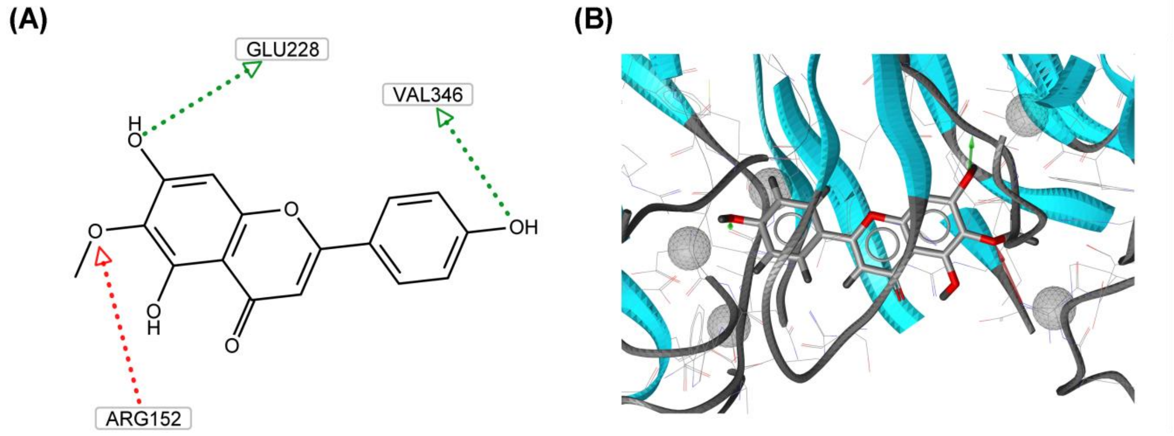

Figure S10. (**A**) Pharmacophore of Inhibitor 10; (**B**) Inhibitor 10 bound to the binding site of the 2009 H1N1 neuraminidase.

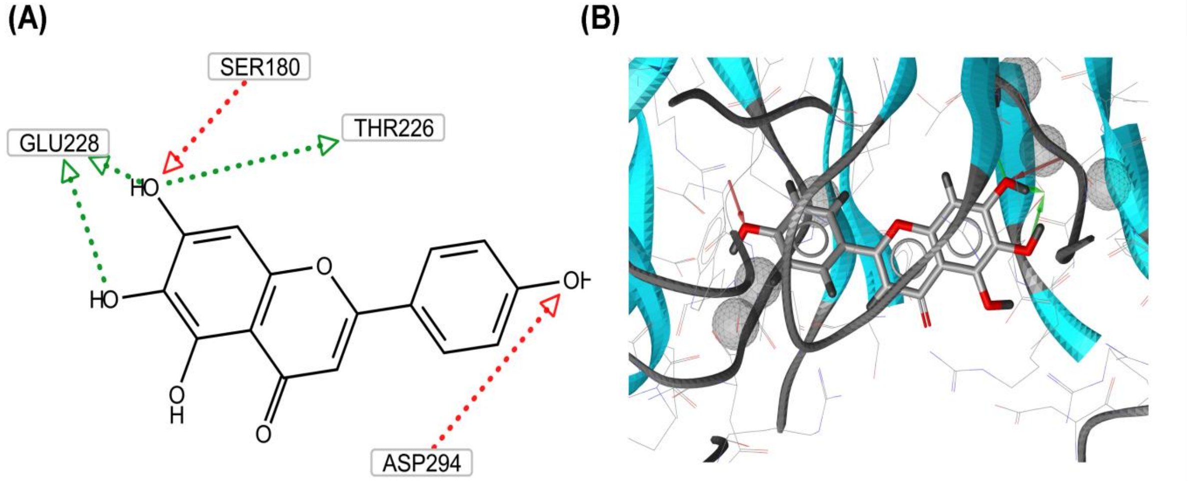

Figure S11. (**A**) Pharmacophore of Inhibitor 11; (**B**) Inhibitor 11 bound to the binding site of the 2009 H1N1 neuraminidase.

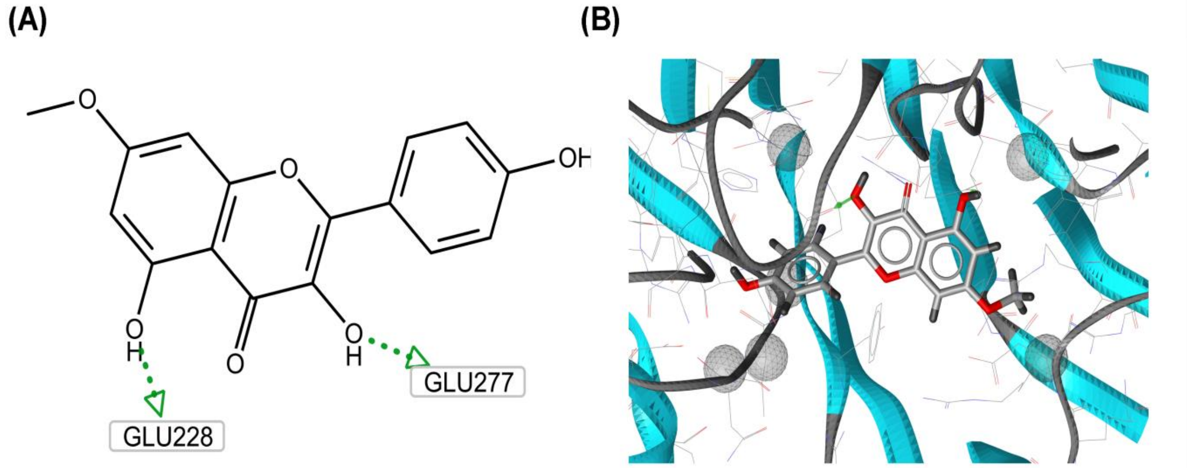

Figure S12. (**A**) Pharmacophore of Inhibitor 12; (**B**) Inhibitor 12 bound to the binding site of the 2009 H1N1 neuraminidase.

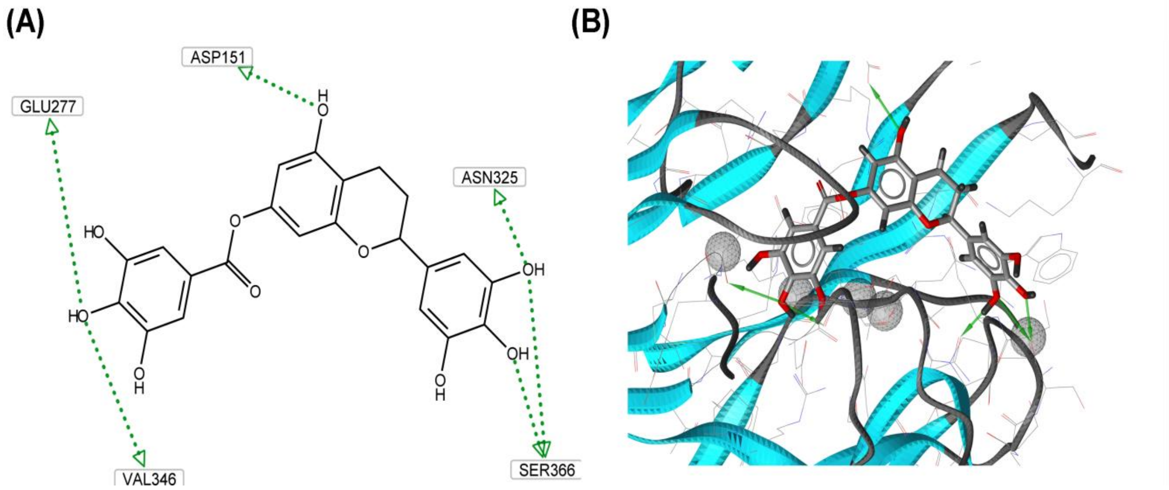

Figure S13. (**A**) Pharmacophore of Inhibitor 13; (**B**) Inhibitor 13 bound to the binding site of the 2009 H1N1 neuraminidase.

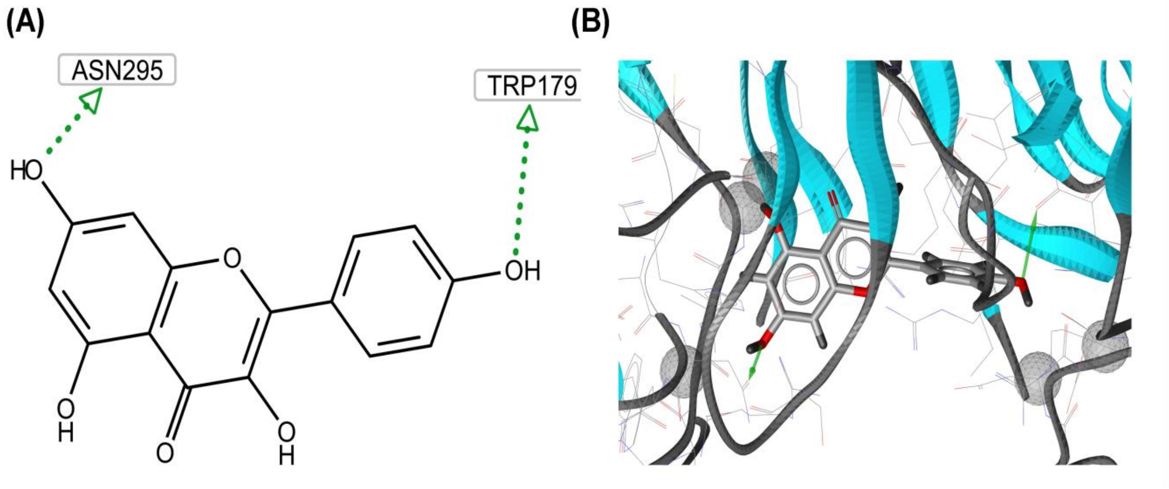

Figure S14. (**A**) Pharmacophore of Inhibitor 14; (**B**) Inhibitor 14 bound to the binding site of the 2009 H1N1 neuraminidase.

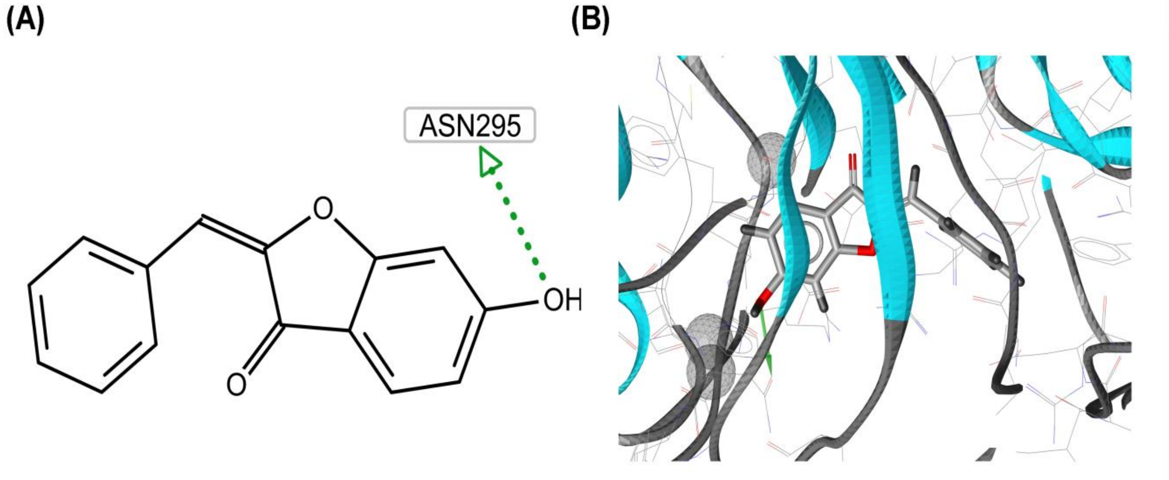

Figure S15. (**A**) Pharmacophore of Inhibitor 15; (**B**) Inhibitor 15 bound to the binding site of the 2009 H1N1 neuraminidase.

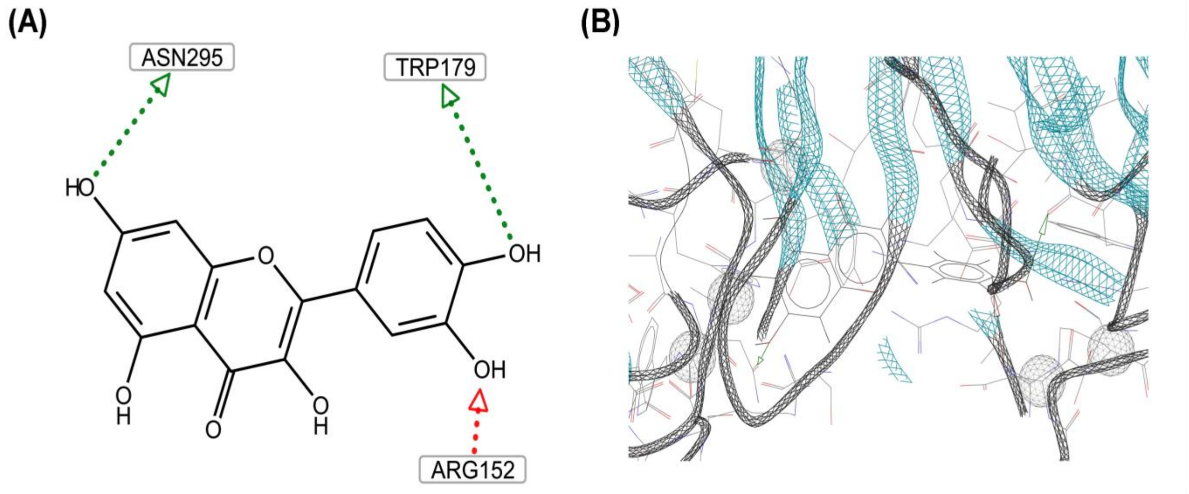

Figure S16. (**A**) Pharmacophore of Inhibitor 16; (**B**) Inhibitor 16 bound to the binding site of the 2009 H1N1 neuraminidase.

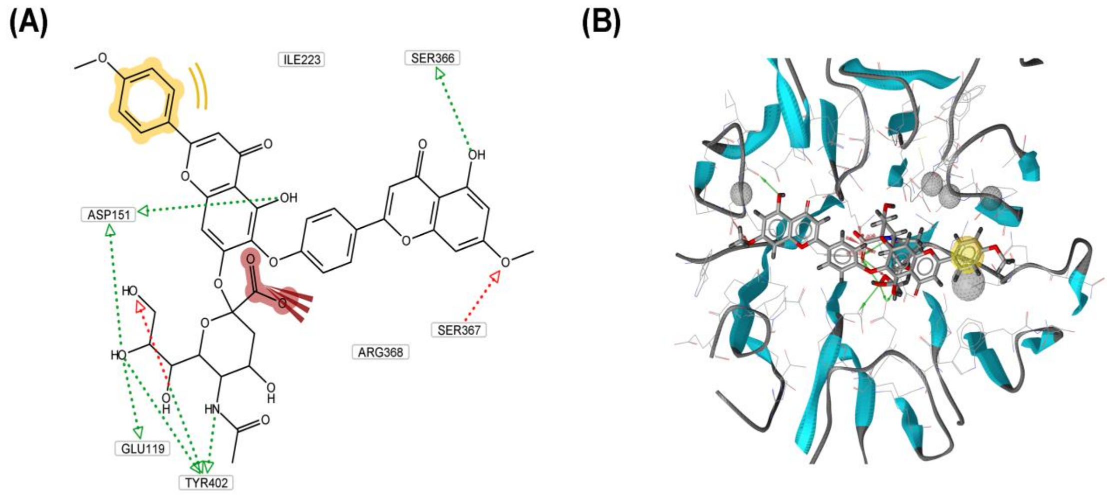

Figure S17. (**A**) Pharmacophore of Inhibitor 17; (**B**) Inhibitor 17 bound to the binding site of the 2009 H1N1 neuraminidase.

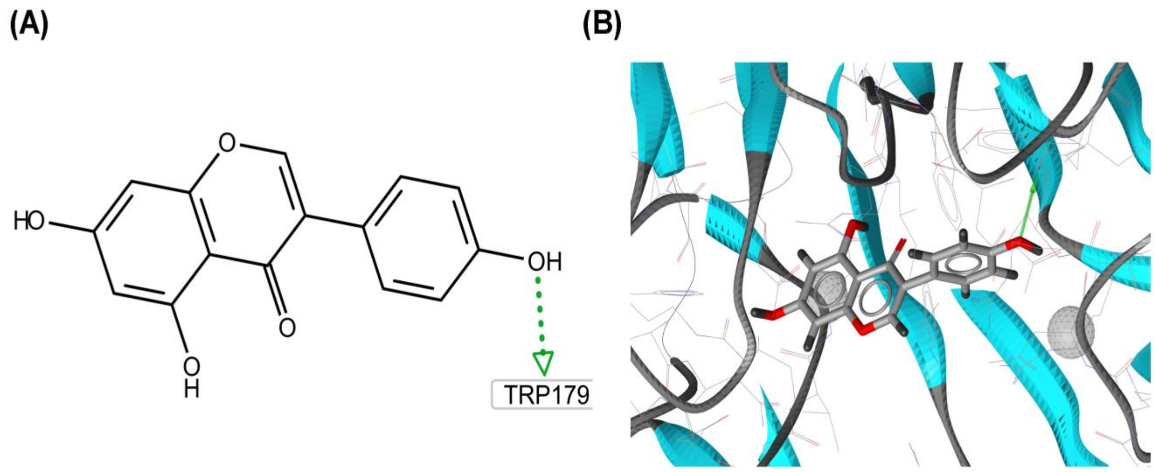

Figure S18. (**A**) Pharmacophore of Inhibitor 18; (**B**) Inhibitor 18 bound to the binding site of the 2009 H1N1 neuraminidase.

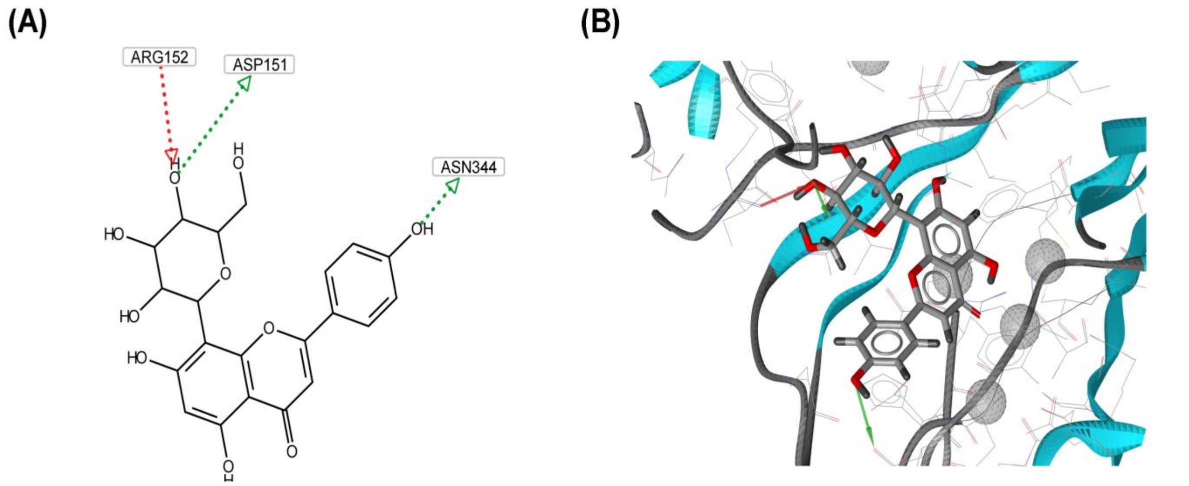

Figure S19. (**A**) Pharmacophore of Inhibitor 19; (**B**) Inhibitor 19 bound to the binding site of the 2009 H1N1 neuraminidase.

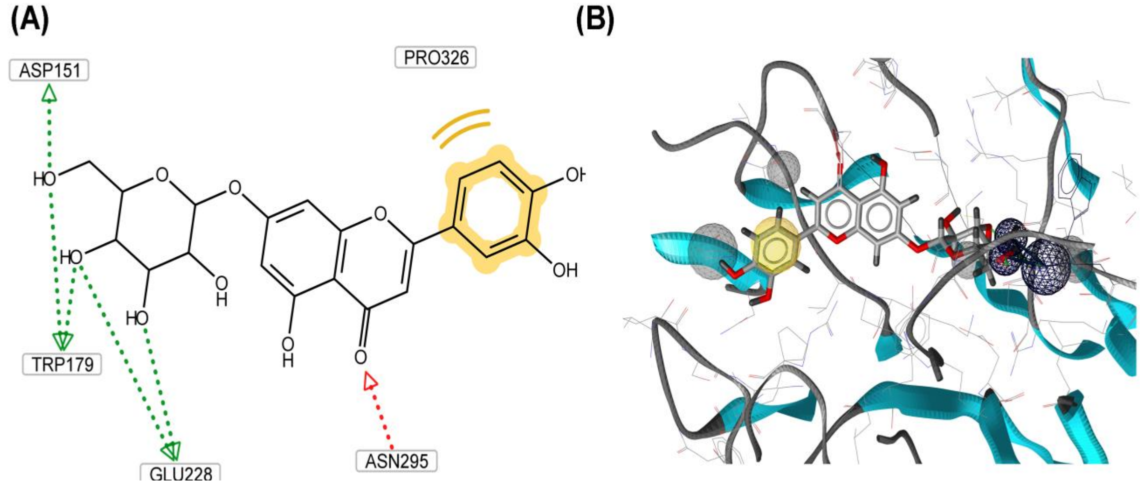

Figure S20. (**A**) Pharmacophore of Inhibitor 20; (**B**) Inhibitor 20 bound to the binding site of the 2009 H1N1 neuraminidase.

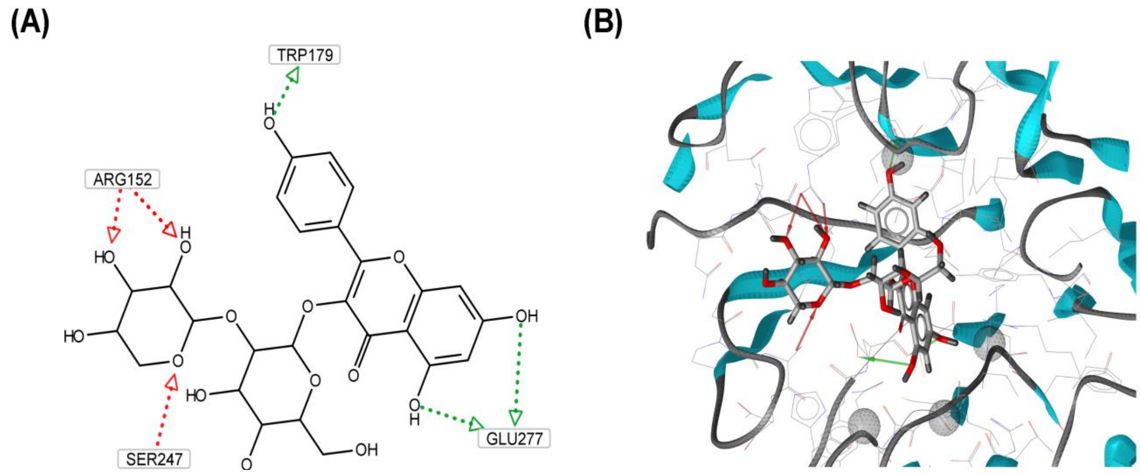

Figure S21. Binding free energies analysis of hydrophobic (aromatic) natures of 20 flavonoids/hydrophobic (aromatic) natures of the important residue regions analyzed by the ligplot program (kcal/mol).

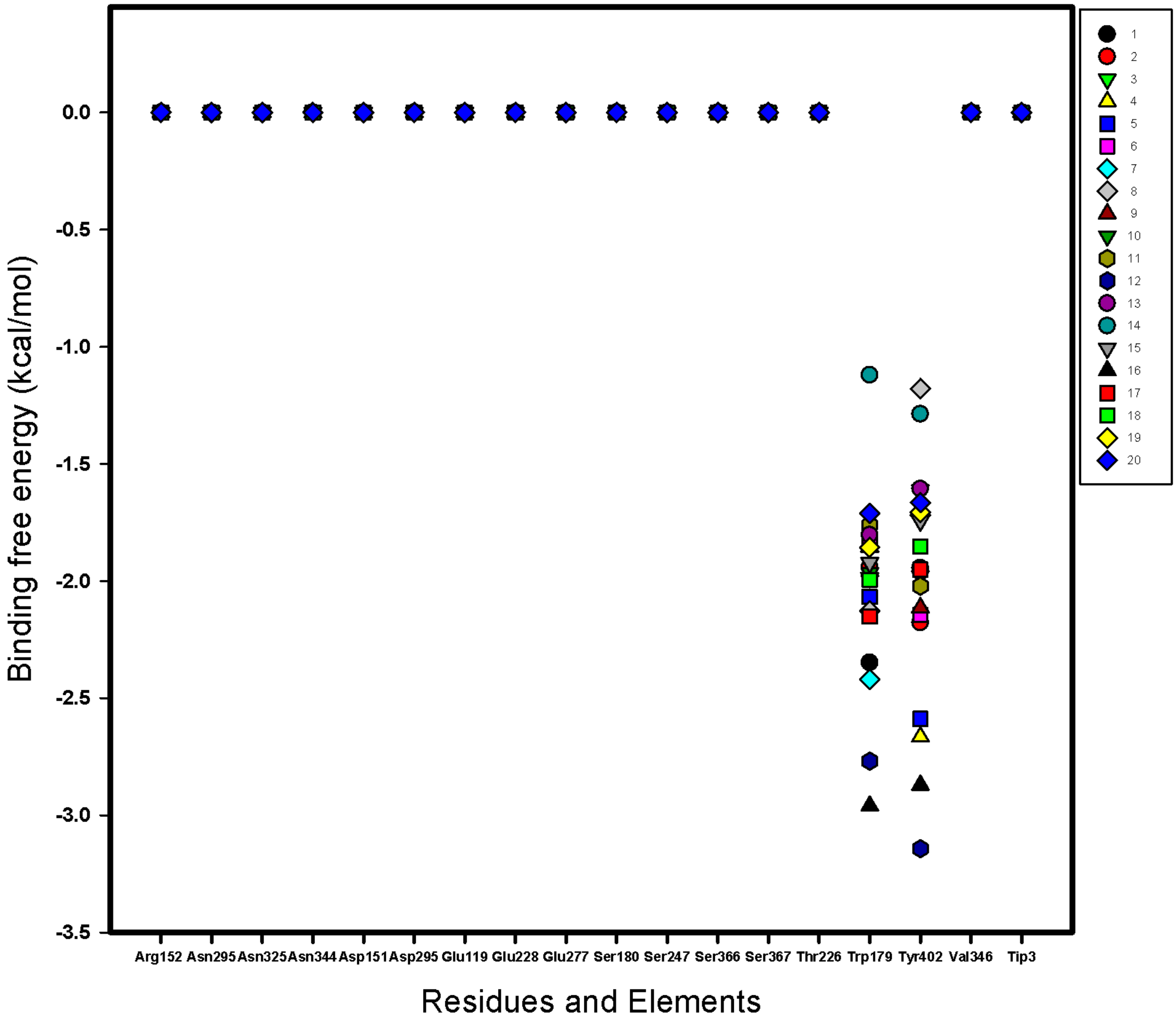

Figure S22. Binding free energies analysis of hydrophilic (–OH, =O, –NH_2 and =NH functional groups) nature of 20 flavonoids/hydrophilic nature of natures of the important residue regions analyzed by the ligplot program (kcal/mol).

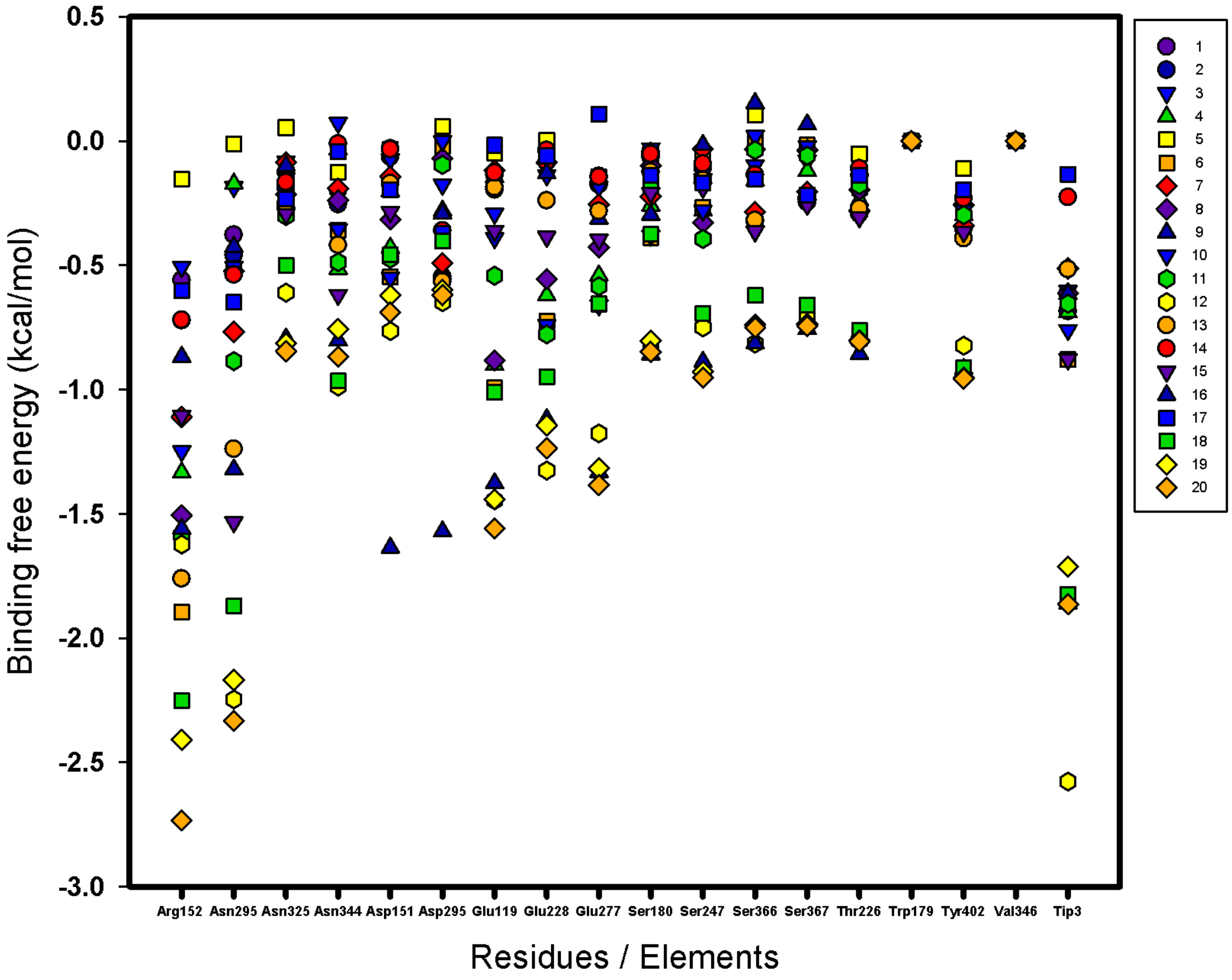

Figure S23. Correlation between binding free energies of Tip3 water molecules, and number of –OH and =O functional group of inhibitors.

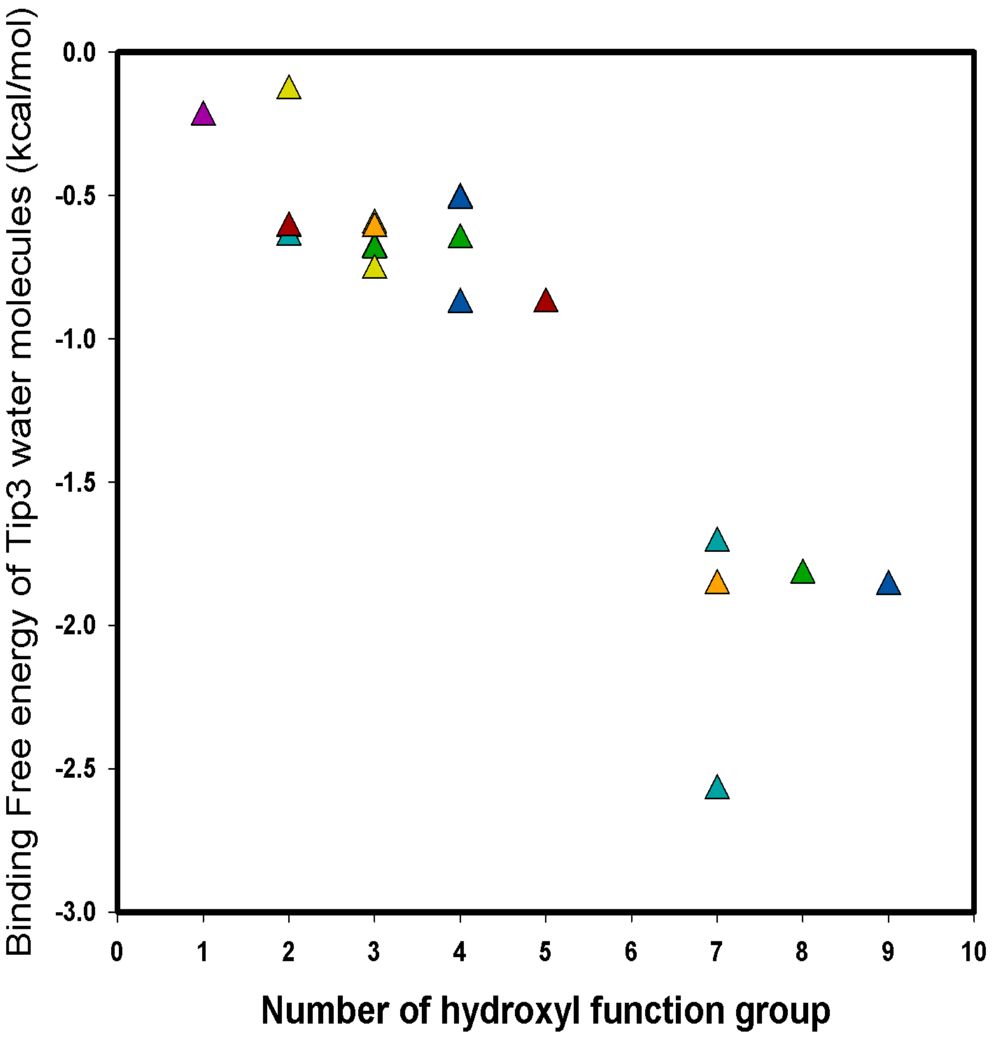

© 2012 by the authors; licensee MDPI, Basel, Switzerland. This article is an open access article distributed under the terms and conditions of the Creative Commons Attribution license (http://creativecommons.org/licenses/by/3.0/).
